# Tricuspid Regurgitation, the Once-Forgotten Valve: Current Management Options and Remaining Gaps After the 2025 European Society of Cardiology Guidelines Update

**DOI:** 10.31083/RCM53100

**Published:** 2026-10-16

**Authors:** Alessandro Cafaro, Amir Tahoun, Marco Mussardo, Alessandro Mandurino-Mirizzi, Luca Quarta, Francesco Rizzo, Antonio Tondo, Fortunato Iacovelli, Dionigi Fischetti, Giuseppe Colonna

**Affiliations:** ^1^Division of Interventional Cardiology, Cardiovascular Department, “V. Fazzi” Hospital, 73100 Lecce, Italy; ^2^Division of University Cardiology, “ICOT” Marco Pasquali Hospital, 04100 Latina, Italy; ^3^Department of Medical-Surgical Sciences and Biotechnologies, Sapienza University of Rome, 04100 Latina, Italy; ^4^Division of University Cardiology, Cardiovascular and Thoracic Department, Policlinico University Hospital, 70124 Bari, Italy

**Keywords:** heart valve diseases, tricuspid valve insufficiency, history of medicine, transcatheter tricuspid valve intervention, edge-to-edge repair, transcatheter tricuspid valve replacement

## Abstract

Historically considered a “neglected” valvular disease, moderate-to-severe tricuspid regurgitation (TR) is now recognized as an important contributor to morbidity, mortality, and right-sided heart failure (HF). Natural history studies show that significant TR is frequently associated with left-sided valve disease, particularly mitral valve disease, and independently predicts poor outcomes as severity increases. The 2025 European Society of Cardiology (ESC)/European Association for Cardio-Thoracic Surgery (EACTS) Guidelines for the Management of Valvular Heart Disease place unprecedented emphasis on TR, elevating this condition from a secondary afterthought to a central element of comprehensive valvular therapy. This reflects an evolution of evidence over the past decade, underscored by increased diagnostic sensitivity through multimodal imaging and the development of novel therapeutic options. However, gaps in the existing evidence persist, and addressing these uncertainties will support future research. This paper provides a comprehensive review of the evidence on TR, incorporates updates from the latest European guidelines, focuses on the most relevant and controversial aspects of patient selection, and identifies key areas where knowledge is insufficient. Furthermore, this review revisits the historical foundations of tricuspid valve research, highlighting how several anatomical concepts fundamental to contemporary interventions were already described as early as the 17th century, underscoring the long-standing scientific interest in this once “forgotten” valve.

## 1. Introduction and Epidemiology

Significant tricuspid regurgitation (TR), defined as moderate-or-greater severity, is a common valvular heart disease (VHD) with an age- and sex-adjusted prevalence of 0.55% in the general population [1]. Its prevalence increases with age and is higher among women; notably up to 4% of individuals aged ≥75 years exhibit clinically significant TR [1]. Globally, 70 million people are affected [2], with an annual incidence of about 300,000 cases in Europe [3]. Isolated TR—defined by the absence of significant comorbidities, structural left-sided heart disease (LHD), pulmonary hypertension (PH) or other overt cardiac etiologies—accounts for 8.1% of significant TR cases and is independently associated with elevated one-year (30–45%) [4] and five-year (60–70%) [5] mortality. Among patients with left ventricular (LV) systolic dysfunction, assessment of TR’s prognostic value is often complicated by coexisting conditions; however, right heart failure (HF) closely correlates with TR severity [6]. Despite these data, only a minority of patients (2.6–10.3%) undergo valve surgery during follow-up [1,5,7], and those managed medically have approximately half the survival rate compared to those receiving surgical intervention (62% vs 35%) [7]. Conversely, valve surgery for significant TR carries considerable in-hospital mortality (10%) and major complication rates (19%), as well as prolonged hospitalization [8,9]. In a large cohort study (N = 10,611), Anand et al. [10] identified five prognostic clusters associated with mortality risk in patients with moderate-or-greater TR. These included: low-risk TR, high-risk TR (linked with PH), coronary artery disease, lung disease and chronic kidney disease (CKD). The highest mortality risk was observed in patients with concomitant CKD or lung disease [10]. Moreover, TR and a peak velocity >2.8 m/s are diagnostic markers of LV diastolic function similarly to increased E/e’ and reduced e’ velocity, so TR represents an indirect marker of elevated LV filling pressures [11].

One of the most notable developments in the 2025 European Society of Cardiology (ESC)/European Association for Cardio-Thoracic Surgery (EACTS) Guidelines for the Management of Valvular Heart Disease is the formal inclusion of transcatheter tricuspid valve interventions (TTVI)—such as edge-to-edge repair and other device-based therapies—with a Class IIa recommendation for symptomatic, high-surgical risk patients with suitable anatomy [12]. Despite improved recognition, advances in diagnostic frameworks, and broader use of innovative therapies, significant gaps in clinical evidence persist—especially regarding the best timing for intervention, methods to quantify right ventricle (RV) dysfunction and long-term results of transcatheter therapies. This review therefore aims to summarize current TR management and identify the principal unresolved questions (Graphical Abstract).

## 2. Anatomy and Function of the Tricuspid Valve

The tricuspid valve (TV) is a complex functional unit consisting of an annulus, usually three leaflets—anterior, posterior, and septal—along with chordae tendineae, papillary muscles (PM) and connections to the right atrium (RA) and RV. It is the most apically placed heart valve and has the largest orifice (7–9 cm^2^) [13,14].

TV anatomy and function were recognized centuries before the modern era. In his *Historia Anatomica* (1606), Andreas Laurenti, physician to King Henry IV of France, clearly described the TV as a structure preventing blood backflow, noting the leaflets’ systolic–diastolic excursions. While cardiac motion remained incompletely understood (“*the nature and cause of its perpetual motion are involved in so many and such great difficulties that they must be considered known only to God and Nature*”), its mechanical function was already clearly appreciated. He based his theories on earlier writings by Galen [15]. In 1757, Albrecht von Haller further refined this understanding by emphasizing the variability in leaflet number (reporting up to five valvular segments), as well as in PM anatomy [16]. This early recognition of morphological heterogeneity anticipated the modern concept of the TV as a dynamic and complex functional apparatus rather than a strictly tri-leaflet structure (Fig. 1, Ref. [15,16]).

**Fig. 1.**
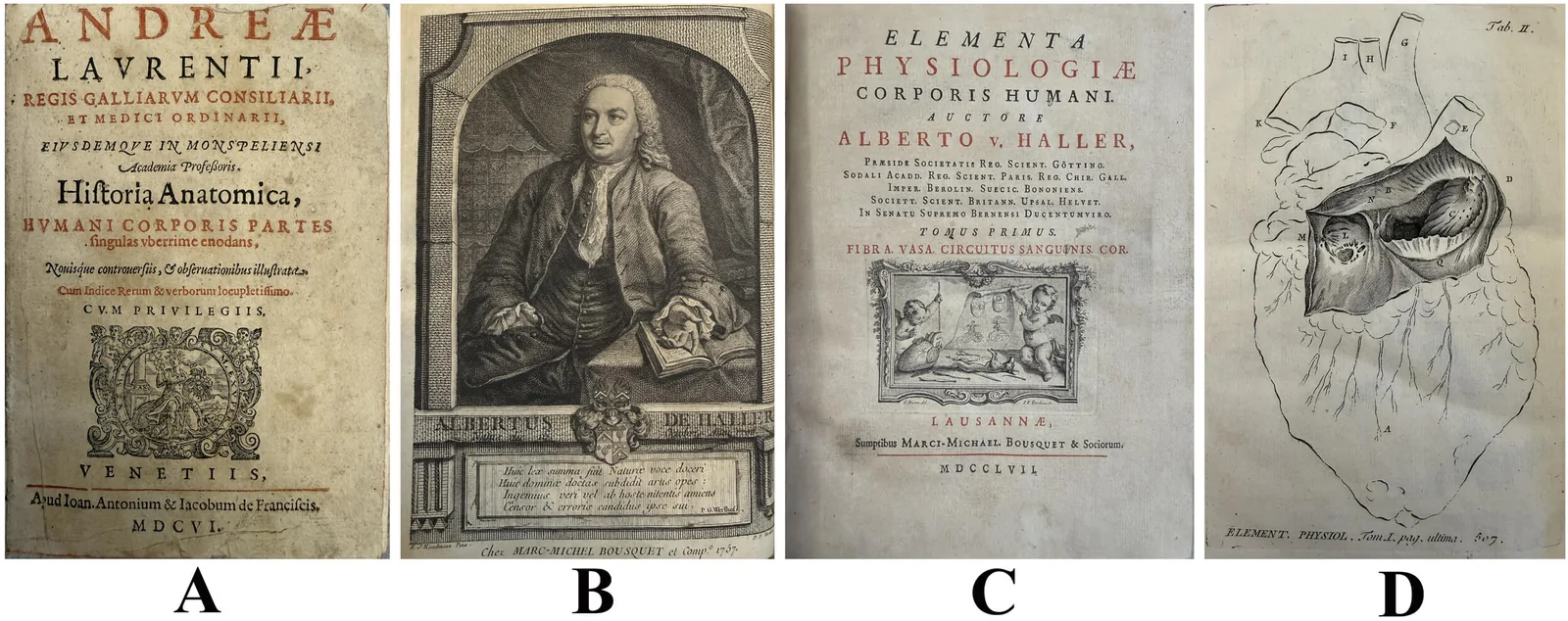
**Historical description of tricuspid valve anatomy variability**. Title pages of *Historia anatomica humani corporis partes* by Andreas Laurenti (1606) [15] (A), and *Elementa physiologiae corporis humani* by Albrecht Von Haller (1757) [16] (C); portrait of Albrecht von Haller (Bern, 1708–1777) (B); engraving og human atrioventricular valves from Haller’s *Elementa physiologiae* corporis humani, Volume I, Table II (D).

Contemporary imaging studies confirm this variability. In a cohort of 120 patients undergoing transcatheter tricuspid edge-to-edge repair (T-TEER), only 48.6% exhibited a three-leaflet morphology, while 5% had a bicuspid configuration; the remaining patients showed more complex leaflet segmentation with up to six identifiable leaflets. Notably, increased leaflet complexity (>3 leaflets) did not significantly impact procedural technical success, despite a trend toward a potential effect [17].

The TV annulus has a dynamic, saddle-shaped form, with its lowest points found at the anteroseptal and posteroseptal commissures. The highest point is where the anterior leaflet attaches to the annulus. Throughout the cardiac cycle, the annulus changes shape: as the ventricles contract, the aortic root pushes against the anterior annulus, while the posterior annulus bends inward toward the septal annulus [18].

Identifying leaflets can be challenging during surgery. The anterior leaflet is typically the largest, the most mobile and is supported by the supraventricular crest. The posterior leaflet may have several scallops and is the shortest circumferentially, while the septal leaflet is the shortest radially, the least mobile and inserts more apically, supported by chordae with direct insertion into the muscle [13,18,19]. The commissure between septal and posterior leaflets, near the coronary sinus (CS), serves as a consistent anatomical landmark, but it may not be immediately apparent. A coaptation reserve of 5–10 mm helps maintain valve function despite annular dilation [13,20].

The TV’s “tensor apparatus” includes two main PM—anterior (usually largest) and posterior—and a variable septal PM, which may be small, multiple, or absent in up to 20% of individuals. The anterior PM supports the anterior and posterior leaflets, while the posterior one supports the posterior and septal leaflets. Chordae may arise directly from the septum, inserting to the anterior and septal leaflets, and thereby anchoring them to the interventricular septum (IVS), while the anterior and posterior leaflets depend on a large anterior PM along the anterolateral RV wall. Besides true chordae (which insert into leaflet tissue), false chordae may connect PM, ventricular walls or both. Most true chordae originate from the apical third of PM, though some arise from the ventricular walls. The normal tricuspid annulus is D-shaped, nonplanar, and has two segments: a larger C-shaped free wall segment and a shorter straight septal segment. In healthy adults, tricuspid annular circumference and area average approximately 12 cm and 11 cm^2^, respectively [21].

Three key structures, at risk during TV procedures, are located near the valve: the noncoronary sinus of Valsalva (SV), which is next to the commissure between the anterior and septal leaflets, making aortic perforation a risk with transcatheter devices anchoring here, the atrioventricular node and bundle of His, which run 3–5 mm behind this commissure at the septal leaflet attachment, so that any pressure or injury at this level can cause heart block, and, finally, the right coronary artery (RCA), which originates from the right coronary SV and travels in the right AV groove to the crux of the heart, helping to indicate the location of the annulus [22].

## 3. Classification of Tricuspid Regurgitation

TR is classified into three categories based on etiology, further refined by integrating the Carpentier classification originally developed for mitral regurgitation (MR): primary TR (PTR), secondary TR (STR), and cardiac implantable electronic device (CIED)-related TR, labeled as lead-associated TR (LTR) [14,23,24].

PTR results from anatomical abnormalities of the TV apparatus and accounts for only 8–10% of TR cases. It is caused by impaired leaflet coaptation due to intrinsic changes such as restricted or excessive leaflet mobility or perforation. All Carpentier types can be observed, and annular, RV and RA dilatation is common. Etiologies include congenital malformations, infective endocarditis, myxomatous degeneration, trauma or post-biopsy injury, carcinoid syndrome, rheumatic disease, radiotherapy and tumors [24].

STR (functional) is more common and typically results from annular dilation associated with RV enlargement and dysfunction, atrial fibrillation (AF), or pressure or volume overload due to PH, often secondary to LHD. Given the heterogeneity in clinical outcomes among patients with STR, a subclassification is necessary [23,24]:

- Atrial type (A-STR): characterized by older age, fewer symptoms, marked annular dilation, reduced tenting height, minimal-to-absent leaflet tethering and preserved leaflet mobility (Carpentier type I). It is associated with significant RA dilation and typically preserved RV volume and function, as well as normal RV-pulmonary arterial (PA) coupling. Etiologies include AF/atrial flutter, advanced age or HF with preserved ejection fraction (HFpEF) [24,25,26].
- Ventricular type (V-STR): marked by significant leaflet tethering, restricted systolic leaflet motion (Carpentier type IIIB), and dilated or dysfunctional TV annulus, RV and RA. Causes include left-sided ventricular or valvular disease, PH, RV cardiomyopathy and RV infarction [24].

A-STR and V-STR have distinct prognostic implications. Patients with A-STR show a significantly lower incidence of death and heart failure hospitalization (HFH) at follow-up [25,26]. While STR severity is the only parameter independently associated with prognosis in A-STR, RV function (in addition to STR severity) has prognostic relevance in V-STR [25]. Nevertheless, A-STR is not a benign condition: A-STR patients remain at increased risk of HFH and excess mortality, particularly in presence of elevated RA pressure, renal dysfunction, AF or combination with MR [14,25,26].

With nearly 800 CIED implantations per million inhabitants annually in Europe [27], LTR has emerged as a distinct clinical entity, accounting for about 5% of severe TR cases [28] and complicating 20–40% of post-procedural courses [29,30]. In many patients the presence of a CIED lead in the context of a TR is incidental (LTR-B or CIED-associated TR). However, TR may be directly attributable to leads traversing the TV and interfering with its function, either when it develops de novo or when a pre-existing TR worsens following RV lead implantation (LTR-A or CIED-related TR) [24,31].

Structural abnormalities of the TV leaflets may be present, and all Carpentier types can occur, often accompanied by annular, RV and RA dilation. Proposed mechanisms include leaflet impingement, chordal entanglement or rupture, leaflet adherence, laceration/perforation or avulsion following lead extraction [24]. Patients with significant CIED-related TR frequently exhibit adverse remodeling of cardiac volumes and function during follow-up, which are associated with worse long-term survival. Moreover, they exhibit a higher risk of HF-related events compared with patients without device-related TR [30,31].

## 4. Tricuspid Regurgitation Imaging Evaluation

2025 ESC/EACTS Guidelines for the Management of Valvular Heart Disease indicate transthoracic echocardiography (TTE) as the first-line imaging modality, as it generally provides sufficient diagnostic information for most patients. TTE enables assessment of TR severity and etiology – including characterization of LHD and, when relevant, evaluation of CIED lead position and its interaction with the TV [23]. It also allows evaluation of RH remodeling and estimation of central venous and pulmonary pressures [12,24]. In candidates for intervention or when detailed anatomical characterization is required (e.g., differential diagnosis between CIED-related and CIED-associated TR), transesophageal echocardiography (TEE), strain imaging, and three-dimensional (3D) echocardiography are recommended [23,24]. Cardiac magnetic resonance (CMR) provides accurate and reproducible assessment of RV size, volumes, and function [12,32]. CMR at 1.5 T has been shown to be safe in CIED evaluation [31,32]. Electrocardiogram (ECG)-gated cardiac computed tomography (CCT) using dedicated right heart (RH) contrast enhancement protocols enables detailed anatomical assessment of RH and RCA, thus being essential for procedural planning and device sizing in transcatheter procedures [12,24].

TR severity is assessed using a 5-grade classification extending beyond “severe” to include “massive” and “torrential” TR, which correlates with symptoms and may improve the evaluation of residual regurgitation following TTVI with prognostic implications [12,24,25,33]. Considering that an effective regurgitant orifice area (EROA) ≥0.4 cm^2^ is linked to increased mortality risk [5], quantitative assessment is recommended. Three quantitative methods are suggested: proximal isovelocity surface area (PISA), planimetry of the 3D vena contracta area (3D-VCA) and quantitative Doppler [25].

Although PISA has been associated with outcomes [34], it may underestimate TR severity compared with 3D-VCA, particularly in the presence of leaflet tethering and low regurgitant jet velocity; these limitations can be partially mitigated by geometric correction [31]. Quantitative Doppler is not yet widely used in clinical practice, but shows good correlation with 3D-VCA [33,34,35]. Finally, TR severity should be assessed under standardized conditions: euvolemic status, stable diuretic therapy, and during end-expiration in spontaneously breathing patients, when alveolar pressure approaches zero, ensuring optimized pulmonary and systemic hemodynamics [11].

## 5. Risk Stratification for Patient Selection

Identification of the underlying cause of severe TR may guide anatomical treatment strategies, but is insufficient for risk stratification. Comprehensive clinical evaluation remains essential to identify patients most likely to benefit from intervention. The recent TRILUMINATE trial, which compared T-TEER with medical therapy, demonstrated consistent improvements in TR severity, NYHA functional class, Kansas City Cardiomyopathy Questionnaire (KCCQ) scores, and 6-minute walk distance (6MWD), despite no significant reduction in mortality and HFH rates at one year [36]. However, the lack of stratification according to baseline risk and residual TR severity may partly explain these neutral findings. Consequently, the 2025 ESC/EACTS guidelines provide a Class I, level C recommendation emphasizing multidisciplinary Heart Team evaluation before intervention, incorporating TR etiology, disease stage (including TR severity, RV and LV dysfunction and PH), surgical risk, and potential for recovery [12,24].

Machine learning algorithms have recently refined risk stratification. Rao et al. [5] identified distinct phenoclusters in patients with severe TR, demonstrating that long-term cardiovascular outcomes, including mortality and HF events, are more closely associated with clinical phenotypes than with etiology alone [10]. Poor prognosis was associated with younger age, RV dysfunction, concomitant moderate-to-severe pulmonic regurgitation, renal impairment, decreased hepatic synthetic function (low serum albumin), and increased red cell distribution width, reflecting systemic congestion and multi-organ involvement (considered two novel predictors of HF outcomes) [37,38]. Notably, worse outcomes were not associated with a higher prevalence of HF with reduced ejection fraction (HFrEF) [5].

Further stratification can be achieved by assessing RV remodeling. In a study by Dietz et al. [39], individuals with STR were divided into four groups based on TTE RV enlargement and function. RV systolic dysfunction was associated with worse outcomes, regardless of the presence of RV dilation [39].

Accurate assessment of RV size and function is therefore central to risk stratification. However, RV evaluation by TTE is challenging due to its complex geometry and lack of well-defined anatomical landmarks. The RV-focused apical four-chamber view reduces measurement variability compared with the conventional LV-oriented view, and foreshortening should be avoided by acquiring the largest basal RV diameter. 3D-echocardiography is recommended in experienced centers when precise volumetric assessment is required [40,41].

RV systolic function requires multiparametric assessment. Tricuspid annular plane systolic excursion (TAPSE) is a simple and widely used measure of longitudinal contractility, with values <17 mm indicating dysfunction [39,42,43]. The RV index of myocardial performance (RIMP) provides global RV assessment but requires accurate timing of isovolumic intervals [40]. Fractional area change (FAC) reflects global systolic function, but it needs the entire RV to be contained in the imaging sector and proper area tracing that includes trabeculae [40]. Tissue Doppler-derived S′-velocity correlates with RV function indices, while speckle-tracking-derived RV free wall strain is clinically feasible but lacks standardized reference ranges [39]. 3D echocardiographic-derived RV ejection fraction (EF) offers improved accuracy, particularly after cardiac surgery, although it remains load-dependent [40]. 3D-echocardiography RV EF can predict cardiovascular outcomes [40] and may outperform TAPSE after TTVI [41]. CMR remains the reference standard for RV volume and EF quantification and is recommended [24,32]. ESC guidelines use conservative cut-off values to avoid overestimating RV function in the context of TR, aiming for early RV dysfunction identification [12,14,39,44].

Assessing PA pressure is crucial to understand TR pathophysiology and rule out precapillary PH [12], which is linked to worse outcomes [45]. TTE may underestimate PA systolic pressure (PASP) in severe TR due to rapid equalization of right-sided pressures [14]. Therefore, RH catheterization (RHC) is recommended in candidates for intervention to evaluate hemodynamics, measure end-diastolic RV pressure, and confirm volume overload [24,45]. Noninvasive RV function measures are often coupled to estimate PA pressure. Notably, the RV-PA coupling ratio (TAPSE/PASP) via TTE offers a simple way to assess how efficiently the RV delivers stroke work to the PA [46,47].

Tello et al. [47] demonstrated that this parameter correlates well with invasive measures of ventricular–arterial coupling (Ees/Ea). In a large cohort of patients undergoing TTVR, Brener et al. [48] showed that TAPSE/PASP provides prognostic information beyond TAPSE or PASP alone. A value <0.406 was a strong predictor of all-cause mortality [48]. Lower coupling ratios reflect inadequate RV contractile reserve relative to afterload, whereas higher pre-procedural values identify patients more likely to tolerate increased pulmonary flow after intervention.

Several risk scores support clinical decision-making. The TRI-SCORE integrates clinical, biological, and echocardiographic variables—including age, NYHA class, signs of right HF, diuretic requirement, renal and hepatic function, LVEF, and RV function—to predict early and mid-term mortality after isolated TV (ITV) surgery [48,49]. In the TRIGISTRY registry, patients with low-to-intermediate TRI-SCORE had significantly better outcomes, whereas high-risk patients derived limited benefit from intervention, with nearly 40% mortality at two years [50]. These findings highlight the importance of early referral, as delayed intervention is associated with worse outcomes, including higher in-hospital mortality and complication rates [8,30].

The TRI-SCORE was developed and validated in surgical cohorts and it is not specific for patients undergoing TTVI. In contrast, the TRIVALVE score was derived in a cohort of patients treated with TTVI [51]. It incorporates five clinical and echocardiographic parameters: AF at baseline, glomerular filtration rate (GFR) <30 mL/min, elevated gamma-glutamyl transferase or bilirubin levels, signs of RH failure, and LVEF <50%. A TRIVALVE score >2.5 identifies patients at high risk for hospitalization and mortality at 12 months. Recently Potratz et al. [52] explored the accuracy of TRI-SCORE, TRIVALVE, and STS score in a population of 457 patients treated with TTVI. The TRIVALVE score showed modest discrimination (area under curve (AUC) 0.60), while STS and TRI-SCORE, despite higher discrimination (AUC 0.68 and 0.72 respectively), were poorly calibrated and tended to overestimate mortality risk [52]. The EuroTR score, developed using artificial intelligence in 1826 patients undergoing T-TEER, evaluates 18 clinical, laboratory, and echocardiographic variables. The EuroTR score showed the highest accuracy (AUC 0.76) for predicting 1-year survival [53]. The EuroTR and TRIVALVE scores are currently the only risk models specifically developed for patients undergoing TTVI.

### Concepts of Proportionality and Remodeling in Secondary Tricuspid Regurgitation

Chronic moderate-to-severe TR imposes RV volume overload that causes progressive RV dilatation, increased wall-stress and dysfunction. This consequently leads to reduced RV output, excessive central venous pressure, renal and hepatic congestion [54]. Concomitant or pre-existing PAH may further aggravate RV failure. Once irreversible cardio-renal and cardio-hepatic syndromes, together with refractory ascites, become established, patients may develop cardiac cachexia, severe functional impairment and increased mortality [55].

Accordingly, the functional and prognostic benefits of TTVI are likely to depend on the ability to achieve durable RV reverse-remodeling following effective TR reduction before irreversible end-organ injury has occurred [56].

A recent observational study enrolled 231 patients, 90% of whom had STR, who underwent serial 3D-echocardiographic follow-up for up to 2 years after T-TEER with TriClip or Pascal [57]. The authors demonstrated that effective post-procedural TR reduction is associated with a biphasic RV reverse-remodeling: (1) An early unloading phase of RV; (2) RV structural reverse-remodeling. Immediately after T-TEER, RV unloading reduced RV end-diastolic volumes (RVEDV) with a slight decline in RV-EF, whereas RV end-systolic volumes (RVESV) remained unchanged. From 6 months onward, progressive reductions in RVESV and continuous improvements in RV-EF and RVEDV resulted in sustained RV structural reverse-remodeling. RVEDV and RVESV improvement peaked at 1-year and remained stable at 2 years. Similar findings were observed for 3D longitudinal strain. Only patients with no-more-than-moderate residual TR experienced RV reverse-remodeling. There was also a durable increase in LV forward stroke volume at follow-up. Patients with no-more-than-moderate residual TR and with RV reverse-remodeling experienced a trend to increased rates of 2-year survival at an exploratory analysis. The prognostic importance of residual TR was also reported in the TRIGISTRY trial [50].

Conversely, correction of massive and torrential TR (50% of currently treated patients) in patients with severe RV dilatation and advanced, potentially irreversible, myocardial damage may not translate into improved survival despite successful reduction of regurgitation [58].

Balancing the severity of valvular regurgitation against the degree of ventricular remodeling has already been shown to influence prognosis in functional mitral regurgitation [59].

Recently Russo et al. [60] conducted a retrospective study evaluating TR quantification with EROA and RV dilatation with RV end-diastolic diameter (RVEDD) in 204 patients of the TRIVALVE registry. Patients were classified according to the EROA/RVEDD ratio into RV-dominant (<0.110), co-dominant (0.110–0.180), and TR-dominant (>0.180) phenotypes.

The RV-dominant phenotype (EROA/RVEDD <0.110), representing disproportionate ventricular disease relative to TR severity, exhibited a trend towards higher mortality at 9.7 months follow-up compared with both co-dominant and TR-dominant phenotypes.

Despite similar procedural TR reduction and comparable TRI-SCORE and TRIVALVE scores across the three groups, patients in the RV-dominant group showed a higher prevalence of mild-to-moderate renal dysfunction and LV impairment. However, after adjustment for baseline characteristics, the prognostic impact of proportionality remained independent of these variables. TR-RV co-dominant phenotype, GFR, and procedural success were the only independent predictors of mortality in adjusted multivariable analysis. Despite similar symptomatic improvement across all phenotypes, consistent with effective TR reduction, the prognostic benefit was confined to the TR-dominant and TR-RV co-dominant groups. These findings should be interpreted cautiously because of the observational design, limited sample sizes, absence of core-laboratory adjudication, and lack of standardized assessment of loading conditions. Both EROA and RVEDD are highly dependent on volume status. These findings support TR proportionality as a promising tool for patient selection, although prospective validation is required. Together with RV reverse-remodeling, this concept may help explain the heterogeneous outcomes of TTVI trials.

## 6. Treatment

TR management has evolved substantially over the past decade and relies on an integrated strategy combining medical, surgical and transcatheter therapies. Treatment should be individualized according to TR etiology, severity, RV function, and comorbidities, ideally within a dedicated multidisciplinary Heart Team. Medical therapy remains the initial cornerstone of management, particularly in STR, and is mainly directed at relieving systemic congestion and optimizing treatment of the underlying conditions—such as LHD, PH, or AF—through diuretics and guideline-directed HF therapy.

According to the 2025 ESC/EACTS Guidelines, when TR becomes severe and symptomatic despite optimal medical treatment, valve intervention is indicated with a class I, level C recommendation. Surgery remains the standard of care in suitable candidates and includes tricuspid valve repair—preferably with annuloplasty—or valve replacement when repair is not feasible [61]. In addition, RV dilation or progressive RV dysfunction should lead to prompt consideration for surgical intervention, even in asymptomatic patients, whereas severe LV or RV dysfunction or severe PH represent contraindications to surgery. Concomitant TV surgery is recommended in patients undergoing left-sided valve surgery with severe TR and should be considered in those with moderate TR and annular dilatation to prevent disease progression [62].

TTVI have rapidly expanded and now represent an important therapeutic option for patients considered at high or prohibitive surgical risk. These approaches include T-TEER, annuloplasty systems, heterotopic caval valve implantation (CAVI), and orthotopic transcatheter tricuspid valve replacement (TTVR). Reflecting the growing evidence supporting their safety and clinical benefit, the latest European guidelines recommend that TTVI should be considered (Class IIa) in symptomatic patients with severe TR who remain unsuitable for surgery despite optimal medical therapy [12]. The therapeutic landscape of TR is rapidly evolving, and optimal management requires careful patient selection based on clinical characteristics, anatomical suitability, and local expertise. Most evidence on TTVI derives from European and North American cohorts. However, as recently highlighted by Hahn et al. [63], despite a similar prevalence of TR, the Asia-Pacific region (APAC) differs substantially in TR etiology, healthcare infrastructure, access to TTVI, and referral patterns. Rheumatic TR remains considerably more prevalent in several APAC countries, reflecting that nearly 70% of the global rheumatic heart disease burden is concentrated in India, China, and Pakistan [64]. These regional differences may influence patient selection, procedural strategies, and outcomes, limiting the generalizability of current evidence. Future multinational studies including underrepresented populations are therefore warranted.

### 6.1 Medical Therapy

Medical therapy has little impact on the progression of TR severity [36]. However, HF represents a frequent and almost inevitable consequence of longstanding severe regurgitation due to chronic RV volume overload and progressive systemic venous congestion. The cornerstone of medical management is guideline-directed HF therapy, primarily aimed at relieving congestion and optimizing hemodynamics [65]. During acute decompensation, pulmonary congestion may also occur, as marked RV dilation and pressure overload can impair LV filling through ventricular interdependence, increasing left-sided filling pressures and venous PH [66]. Since the magnitude and speed of the diuretic response significantly influence outcomes in acute HF, with more effective decongestion associated with lower risk of rehospitalization and mortality [65,66,67], diuretics play a pivotal role both during and after acute phases. Treatment usually starts with loop diuretics and with escalation, when necessary, to mineralocorticoid receptor antagonists, thiazide diuretics, and sodium–glucose cotransporter-2 inhibitors (SGLT2i) to achieve adequate decongestion [68].

Associated conditions such as PH and AF should be managed appropriately, including pulmonary vasodilators for selected types of PH and rhythm-control for AF [69,70]. In addition, treatment with SGLT2i as part of guideline-directed HF therapy has been associated with an early reduction in PAP after three months [71], together with improvements in RV function and TR severity [68,71]. Although mechanisms remain unclear, these findings suggest that SGLT2i may contribute to pulmonary decongestion beyond their diuretic effect and may represent a promising adjunctive therapy in appropriately selected patients. Dedicated trials are needed to clarify these findings.

Although medical therapy is essential for symptom relief and congestion control, surgical or transcatheter intervention remains necessary to improve outcomes in patients with severe symptomatic TR [70,71].

### 6.2 Surgical Treatment

Tricuspid valve procedures are relatively uncommon, accounting for only 3% of cardiac surgical procedures [72]. The 2025 ESC/EACTS Guidelines differentiate between patients requiring surgery for LHD and those undergoing ITV intervention. In the former group, concomitant TV surgery carries a Class I recommendation. In the latter group, surgery is recommended for symptomatic patients with severe PTR [12]. These indications must be interpreted in light of the substantial operative risk associated with ITV surgery because ITV replacement has been associated with poor outcomes: 12% in-hospital mortality and a 31% rate of major complications. Moderate-to-severe RV dysfunction was identified as an independent predictor of adverse outcome [72,73]. By contrast, ITV repair seems to be associated with lower adjusted mortality and morbidity than replacement, especially when performed on the beating heart [74]. Surgical treatment can therefore be broadly divided into TV repair and replacement.

#### 6.2.1 Tricuspid Valve Repair

TV is traditionally considered challenging to repair because of its dynamic saddle-shaped annulus and variable leaflet morphology. Early repair techniques included suture annuloplasty (De Vega technique) and commissuroplasty with creation of a functionally bileaflet valve (Kay technique). Although these approaches provided temporary symptom relief, their long-term results were unsatisfactory [75].

Modern annuloplasty techniques use bands and rings. Patients receiving ring annuloplasty have up to 82% freedom from recurrent TR after 5.7 years, compared to 39% with suture-only methods. Early moderate-or-greater TR recurrence occurs in 14–23% of cases, while severe recurrent TR is observed in up to 10%. Better long-term outcomes have also been demonstrated, at 15 years of follow-up, compared with suture techniques [76].

The primary target of annuloplasty is the compression of the annular segment included between the RV free wall and the supraventricular crest as the classical asymmetric annular dilation observed in TR is determined by the dilation of these structures while the IVS remains fixed. Indeed, leaflet remodeling is not uniform: the posterior leaflet undergoes the greatest elongation, reaching up to 80% of its annular length [77]. Additionally, when the annulus expands, it not only increases in size but also may flatten, losing its original saddle shape. Commercial tricuspid rings or bands are designed to accommodate these geometric changes, ranging from flat designs to those with elevation profiles intended to restore normal tricuspid annular height. Both rigid and semi-rigid devices are utilized to stabilize the annulus during systole, whereas flexible bands aim primarily to mitigate annular dilatation. Device sizing is based on the length of the septal leaflet base and the surface area of the leaflet tissue. The most common suture technique involves interrupted annular stitches with braided suture material. It should be avoided to injure the conduction system and the aortic root when placing stitches near the septal and anterior leaflet. Sometimes, an additional surgical step is required, especially in cases of severe tethering to obtain a more durable repair. It may consist either of an enlargement of the anterior leaflet, by replacing it with a portion of autologous pericardium, or of the so-called “clover technique”, in which the valve leaflets are sutured together at the midpoint of their free edges, followed by implantation of a semi-rigid ring [78,79,80].

#### 6.2.2 Tricuspid Valve Replacement

If the valve is severely compromised, replacement is generally preferred. The procedure is performed either on a beating heart or under cardioplegic arrest, depending on surgeon preference, clinical setting and the patient risk profile. A beating-heart approach may be favored in high-risk or redo procedures to minimize myocardial injury.

Bioprosthetic valves are often considered the preferred option for right-sided valve replacement. In this setting, the risk of valve thrombosis is higher than in the left heart due to lower pressure conditions and reduced levels of prostacyclin-mediated antiplatelet activity. Nevertheless, bioprosthetic valves tend to demonstrate greater durability in the tricuspid position, likely as a result of the lower hemodynamic stress [81].

Mechanical prostheses may be considered in selected cases, such as in patients with small annular size or in those who already require long-term anticoagulation.

The surgical technique involves excision of the native leaflets—usually preserving the septal leaflet—and implantation of the prosthesis at annular level. Accurate prosthesis positioning is mandatory to avoid obstruction of the RV inflow or outflow tract.

TV replacement is frequently performed as a redo-operation following previous repair and often involves patients with advanced disease and high surgical risk. Accordingly, outcomes remain suboptimal, with reported intraoperative mortality rates of up to 18% in some series [79].

In a population of patients undergoing surgery (N = 426), 30-day mortality rates were 7.6% and 3.9% for TV replacement and TV repair, respectively (*p* = 0.1). TV replacement was also associated with a higher risk of permanent pacemaker implantation (13.5% vs 4.9%, *p* = 0.002) and re-exploration for bleeding (13.3% vs 6.8%, *p* = 0.02). Pericardial effusion and stroke were more frequent following TV replacement compared with repair, however, these differences did not reach statistical significance [9].

### 6.3 Transcatheter Tricuspid Valve Interventions

One of the most important updates in the new 2025 ESC guidelines on valvular heart disease compared to the 2021 version is the promotion of TTVI techniques from a class IIb recommendation with level of evidence C to a class IIa recommendation with level of evidence A for the treatment of severe TR in symptomatic high-risk patients without concomitant LHD requiring surgery, who do not respond to medical therapy, and in the absence of severe RV dysfunction or severe precapillary PH [12]. This reflects the rapid technological development of these procedures and the large body of evidence in recent years. The main percutaneous techniques include T-TEER, direct annuloplasty, and orthotopic and heterotopic TV replacement (Fig. 2).

**Fig. 2.**
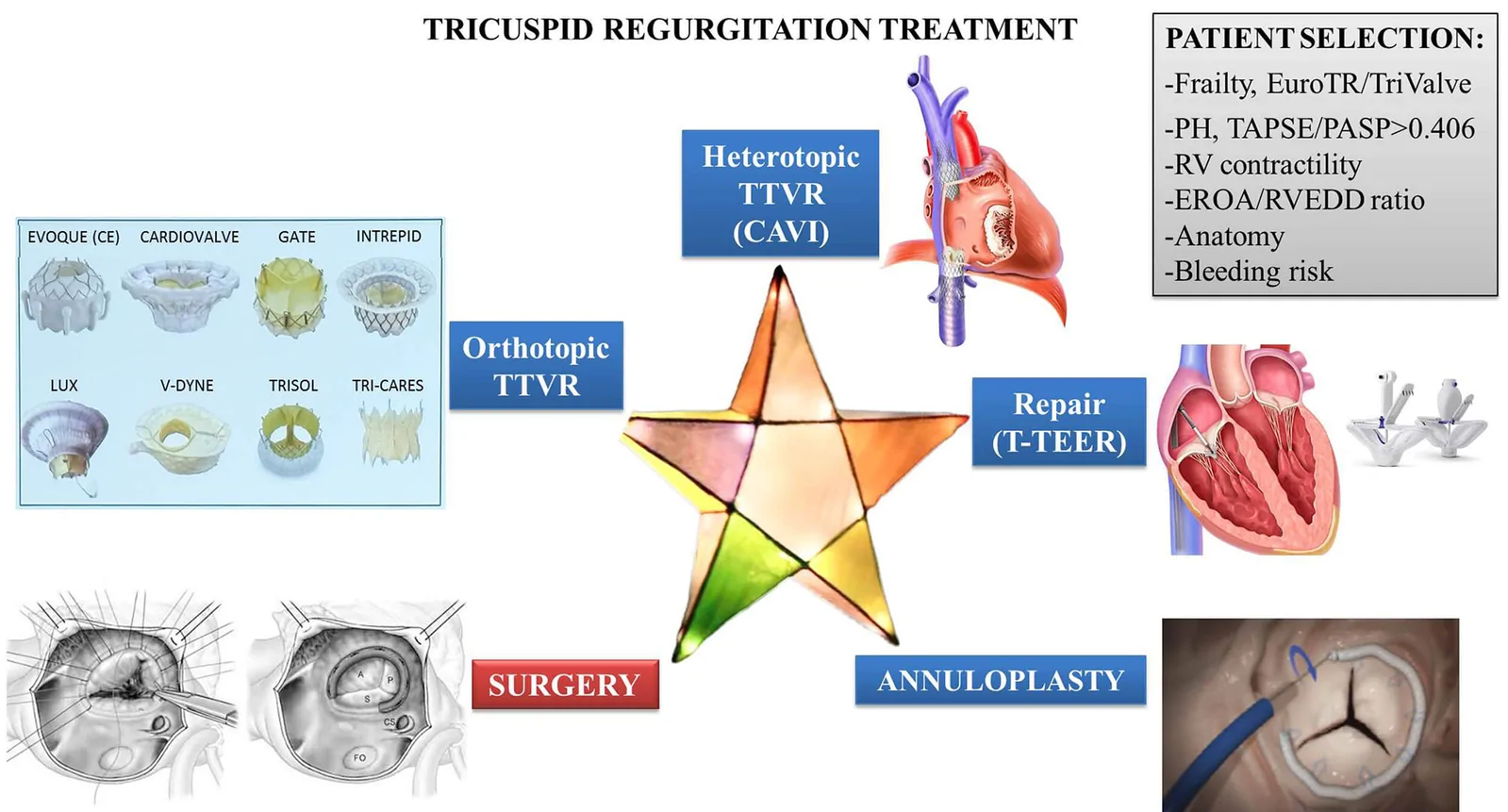
**Treatment strategies for tricuspid regurgitation and key determinants of patient selection**. Overview of current therapeutic options, including surgical intervention (shown in red) and transcatheter options (shown in blue): transcatheter tricuspid edge-to-edge repair (T-TEER); orthotopic transcatheter tricuspid valve replacement (TTVR); heterotopic caval valve implantation (CAVI), and transcatheter annuloplasty. Key factors influencing treatment selection are also illustrated, including frailty, EuroTR and TRIVALVE scores, pulmonary hypertension (PH), right ventricular (RV) function (e.g., TAPSE/PASP ratio), tricuspid regurgitation proportionality (e.g., EROA/RVEDD ratio), anatomical suitability and bleeding risk.

#### 6.3.1 Edge-to-Edge Repair

T-TEER reduces TR by approximating valve leaflets with one or more clips, though outcomes are closely tied to anatomical suitability. Two different procedural strategies, bicuspidalization and triple-orifice approach, showed comparable peri-procedural outcomes, with paramount importance of grasping the septal leaflet in order to combine the coaptation gap reduction to an annuloplasty-effect [82]. A retrospective analysis of 491 patients screened for TTVI showed that about 32% were excluded due to large coaptation gaps and non-central jet locations—confirming these as key predictors of procedural feasibility and success [83].

T-TEER is the most established transcatheter repair strategy, supported by growing evidence from trials and real-world registries. The two main dedicated systems are TriClip (Abbott Cardiovascular), specifically designed for TTVI, and PASCAL (Edwards Lifesciences), which can be used for both atrioventricular valves. These devices come in various sizes to accommodate different anatomical configurations (Fig. 3).

**Fig. 3.**
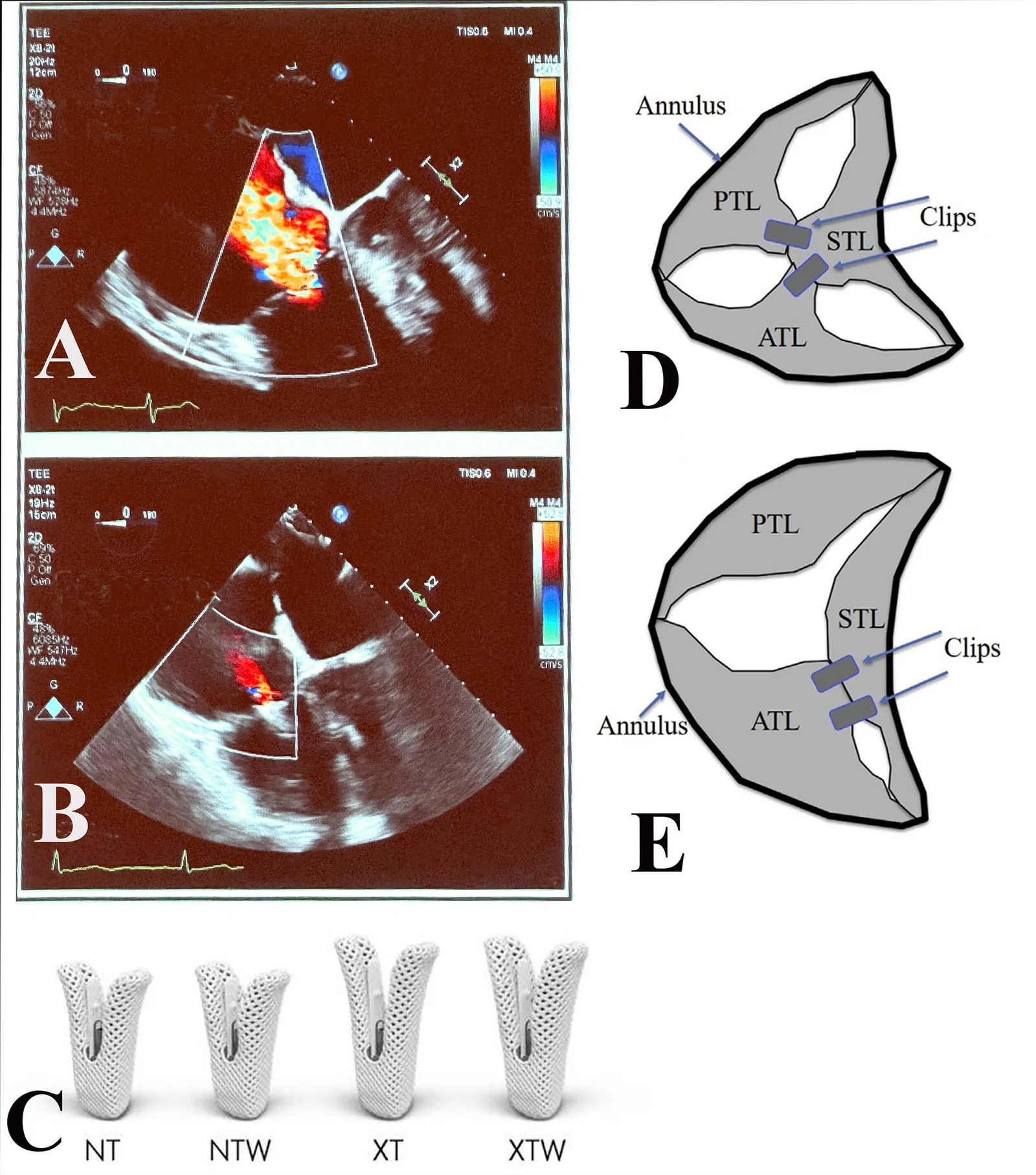
**Transcatheter tricuspid edge-to-edge repair (T-TEER)**. Severe TR before (A) and after (B) TriClip implantation; TriClip device sizes (C). Schematic representation of T-TEER techniques, including triple-orifice (D) and bicuspidalization approaches (E), highlighting clip positioning relative to the anterior, septal (STL), and posterior leaflets and the tricuspid annulus. Traction of the STL leaflet provides an annuloplasty effect.

The TriClip system, particularly in its latest generation, incorporates features such as independent leaflet grasping and real-time pressure monitoring, improving procedural control and adaptability. In the bRIGHT post-approval study [84], which enrolled a high-risk population with advanced symptoms, in-hospital mortality and adverse events were very low (≈1% and 2.7%, respectively), with procedural success achieved in 77% of cases and early improvements in symptoms and quality of life (QoL). Similarly, the TRILUMINATE trial [85,86] (both single-arm and randomized phases) demonstrated substantial TR reduction—with 87% of treated patients achieving moderate-or-less TR at 30 days in the randomized cohort—along with sustained clinical benefits and low complication rates up to 2 years. To date, TRILUMINATE is the randomized clinical trial (RCT) with the longest published follow-up. Conducted across 65 centers in Europe and the United States, it enrolled 350 patients with severe symptomatic TR at intermediate-or-higher surgical risk and no other valvular disease requiring intervention. Patients were randomized to T-TEER with TriClip plus medical therapy or to medical therapy alone. Torrential TR was present in 50.9% of patients and approximately 90% had STR. Mean baseline EROA/RVEDD was 0.12, while TAPSE <17 mm was observed in 52% and 59% of the T-TEER and control groups, respectively. Conducted during the SARS-CoV-2 pandemic, the trial recorded fewer clinical events than anticipated. The hierarchical primary endpoint (all-cause death or TV surgery, HFH, and KCCQ improvement) favored T-TEER at 1-year, mainly driven by improved health status, whereas mortality and HFH were initially similar between groups. At 1 year, NYHA class improved in 83.9% of T-TEER patients versus 59.5% of controls, and 6MWD was also greater in the intervention group. Single-leaflet device attachment and major bleeding occurred in 7.0% and 4.7% of patients.

Longer-term follow-up confirmed sustained symptomatic benefit, RV reverse-remodeling, and reduced HFH.

Additional support comes from the TRIS study [87], confirming improved outcomes in severe TR, and bRIGHT registry [84,88,89], demonstrating consistent real-world performance at 1 year.

The PASCAL system represents an alternative approach, featuring broad paddles, independent clasps, and a central spacer designed to reduce leaflet stress and fill the regurgitant orifice. In the CLASP TR trial [90,91], 85% of patients with successful implantation achieved at least a one-grade TR reduction at 30 days. At 1-year, TR reduction remained sustained, accompanied by high survival rates and low HFH. These data are supported by real-world registries and a large observational study including more than 1000 patients, in which 83% achieved TR reduction at 1 year with significant clinical improvement [92,93]. Newer device iterations, such as PASCAL Precision, have been associated with shorter procedures and higher procedural success [92]. The ongoing CLASP II TR randomized trial is expected to further clarify long-term outcomes compared with medical therapy [94].

Earlier T-TEER experience relied heavily on off-label use of the MitraClip system. These studies demonstrated that approximately 90% of patients achieved at least a one-grade reduction in TR at 6 months, with durable effects at 1 year, thereby establishing the feasibility of T-TEER before dedicated devices became available [95,96]. Novel systems such as DragonFly have also been explored, with early case-based evidence suggesting feasibility and safety, although data remain limited [97].

Beyond device performance, patient phenotype also influences T-TEER outcomes. Data from the TRIVALVE registry [96] showed that patients with A-STR had better 1-year survival compared to those with V-STR, particularly in cohorts predominantly treated with MitraClip. This observation was reinforced by the EuroTR registry, which demonstrated greater TR reduction, lower symptom burden, and higher survival free from HF hospitalization (HFH) at 2 years in patients with A-STR [98]. Similarly, Scheffler et al. [99] reported comparable procedural success between TriClip and PASCAL, but lower rates of death and rehospitalization among A-STR patients compared with those with V-STR. A-STR, accounting for 30% of all real-world patients treated with T-TEER, were younger, with lower STS, TRI-SCORE, and comorbidities, reported also less advanced biventricular HF and better hemodynamic profile than non-atrial STR. Lower risk profile is probably one of the reasons of better study outcomes. While A-STR patients had excessive RA dilatation and preserved RV dimensions and contractility, non-atrial STR had wide annular diameters and leaflet tenting linked with worse TR and lower procedural success. A-STR patients were probably treated at an earlier stage of TV disease, when there is only mild ventricular dysfunction. In addition, the predominance of annular dilatation with relatively preserved leaflet mobility and RV function makes A-STR anatomically more suitable for T-TEER repair than V-STR, in which severe leaflet tethering and ventricular remodeling often limit procedural success.

Overall, RCTs, single-arm studies, and large registries consistently show that T-TEER—using both TriClip and PASCAL systems—achieves meaningful and sustained reductions in TR severity with a favorable safety profile. At the same time, anatomical factors and underlying TR etiology remain critical in determining both procedural success and long-term clinical outcomes.

#### 6.3.2 Annuloplasty Devices

TTVI also includes annuloplasty systems, which most closely reproduce surgical repair by reducing annular dilation while preserving native valve anatomy and keeping future treatment options open. Despite technological progress, patient selection remains a major limitation. In a cross-sectional study of 547 patients, nearly 42% were not eligible for percutaneous repair, mainly due to anatomical constraints such as severe annular, atrial, or RV dilation, followed by clinical futility and technical challenges [100].

Cardioband is currently the only annuloplasty device approved in Europe and the most extensively studied. It is a direct ring-based system implanted via transfemoral venous access, in which multiple anchors are placed along the annulus and progressively tightened, under imaging guidance, to achieve annular reduction. Its clinical performance has been evaluated in several studies. The first feasibility study (N = 37) reported 1-year consistent reduction of TR with QoL and symptom improvement [101]. The TRI-REPAIR trial, a single-arm study in patients with functional TR, demonstrated sustained efficacy, with approximately 72% of patients achieving moderate-or-less TR at 2 years, together with significant improvements in 6MWD and QoL [102,103]. These findings were supported by the TriBAND study, where 69% of patients achieved TR reduction below moderate severity at 30 days, and by a U.S. early feasibility study showing favorable 1-year clinical outcomes, including high survival and low HFH rates [104]. A meta-analysis further confirmed that Cardioband promotes reverse-remodeling and has a good safety profile, although procedural complexity and the risk of coronary artery injury due to anchor proximity limit its use to highly experienced centers.

Other annuloplasty devices are under investigation or in early clinical use. The K-Clip system, which reduces annular dimensions through a clamp-based mechanism, has shown promising results. In an initial clinical study, successful implantation was achieved in all patients, with 93% experiencing TR reduction at 30 days [105]. A larger prospective trial with 96 patients confirmed high technical success and sustained TR improvement at 1 year, along with evidence of RV reverse-remodeling and excellent survival free from HFH [106]. Subgroup analyses, such as patients with AF RV–PA uncoupling, have also demonstrated functional and clinical benefits [107,108].

Similarly, the TriCinch system, which applies tension between an annular anchor and a stent in the inferior vena cava to reshape the annulus, showed encouraging early results in the PREVENT trial, where most patients achieved at least a one-grade reduction in TR without major procedural complications [109]. In contrast, the TRAIPTA approach, which reduces annular size externally via a pericardial implant, remains at the preclinical stage but has demonstrated proof-of-concept efficacy in animal models [110].

Clinical outcomes after annuloplasty may also differ according to TR etiology. Preliminary data suggest that patients with A-STR may derive greater benefit compared to those with V-STR. For instance, studies on Cardioband reported similar procedural efficacy between these groups but indicated a higher 1-year survival in A-STR patients, along with a greater degree of TR reduction over time. However, results remain somewhat inconsistent, particularly regarding HFH, highlighting the need for further investigation [111].

Overall, transcatheter annuloplasty represents a physiologically appealing strategy with growing clinical evidence supporting its safety and effectiveness. Nonetheless, anatomical constraints, procedural complexity, and variability in patient response continue to limit its widespread adoption.

#### 6.3.3 Transcatheter Tricuspid Valve Replacement

TTVR has emerged as an alternative to repair techniques, aiming to eliminate TR by replicating surgical valve replacement while avoiding open-heart surgery. These systems may overcome some anatomical limitations of repair procedures and are theoretically applicable even in complex scenarios such as CIED-related TR. However, patient selection remains challenging. In a retrospective study of 327 patients undergoing pre-procedural imaging, nearly two-thirds were unsuitable for TTVR, mainly due to excessive annular dilation (>53 mm), severe RV dysfunction or unfavorable leaflet anatomy [100].

Early TTVR approaches involved valve-in-valve and valve-in-ring procedures using balloon-expandable prostheses originally designed for the aortic or pulmonary position. These strategies effectively treated failed surgical repairs. In the VIVID registry, including 306 patients, three-year outcomes were acceptable, with mortality, reintervention, and valve-related complication rates of 17%, 12%, and 8%, respectively [112,113]. These procedures can also be performed in patients with pacing leads without mandatory extraction, although procedural success is highly dependent on imaging, anatomical selection and device sizing.

TTVR devices are broadly classified into orthotopic valves, implanted at the native tricuspid annulus, and heterotopic systems, positioned in the venae cavae to reduce venous congestion (CAVI).

Among orthotopic devices, the EVOQUE system (Fig. 4) currently represents the most robustly studied technology and the only system currently approved in Europe [114]. In the TRISCEND trial (172 patients), cardiovascular mortality was low (1.7% at 30 days, 9.4% at 1 year), with significant improvements in symptoms, functional status, and QoL, despite a non-negligible rate of adverse events and pacemaker implantation [115,116,117]. Earlier studies and compassionate-use experiences reported high procedural success (≈92%) and near-complete TR reduction in the vast majority of patients at follow-up. The TRISCEND II randomized trial, comparing EVOQUE with medical therapy, showed markedly higher rates of TR reduction in the intervention group, although some methodological limitations (e.g., reliance on soft endpoints) have been noted [118]. Compared to T-TEER there is a higher risk of major bleeding (28-F versus 24-F sheaths) and of infections. Durability data are lacking, but future valve-in-valve treatment is theoretically feasible [114].

**Fig. 4.**
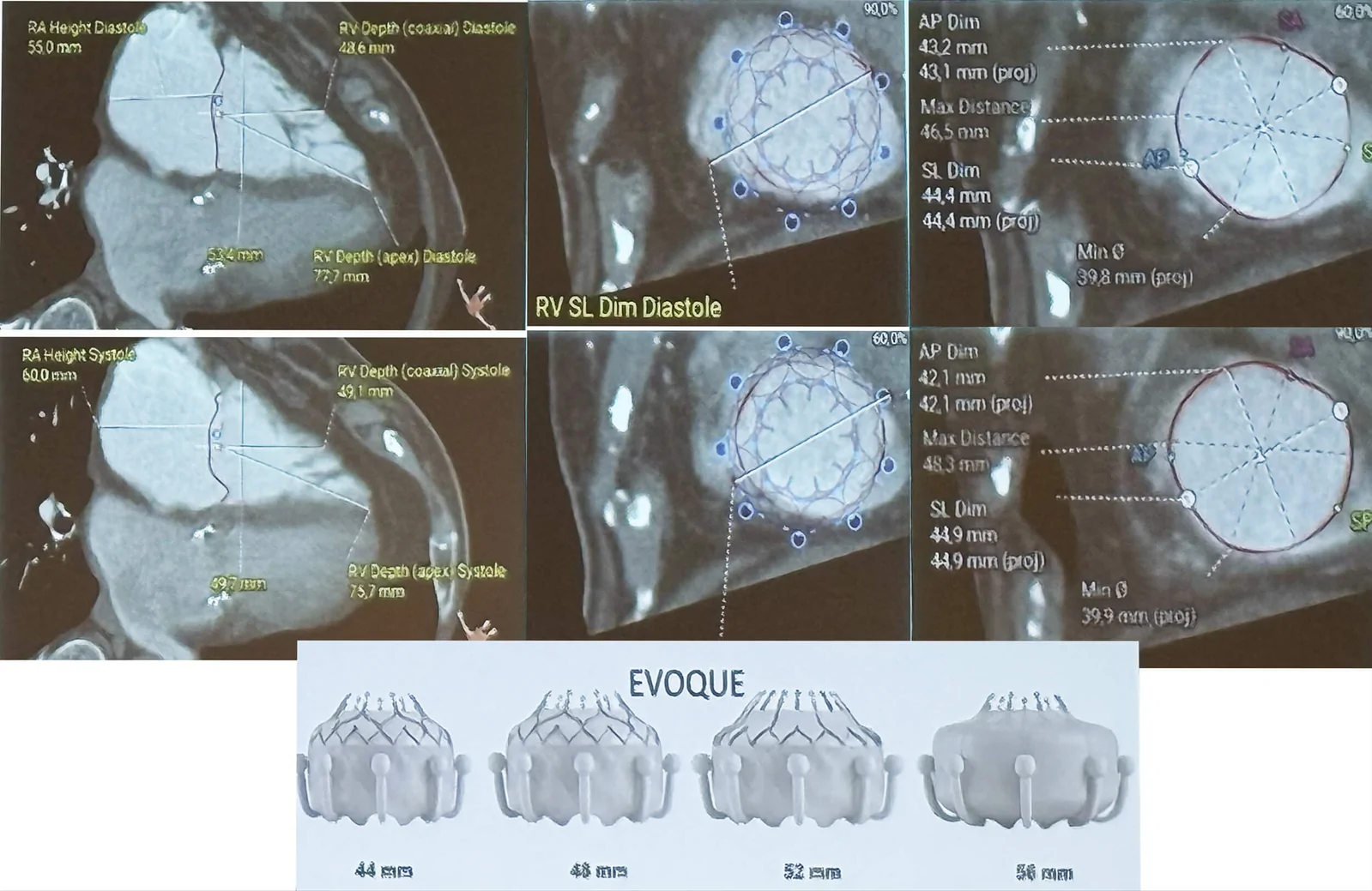
**Orthotopic transcatheter tricuspid valve replacement (TTVR) planning with the EVOQUE device**. Multiplanar CCT reconstructions of the TV apparatus and RH chambers. Upper panels show detailed assessment of the TV annulus in systole and diastole, including anteroposterior, septolateral, maximum, and minimum diameters, annular area, and perimeter, highlighting its dynamic geometry. Basal RV dimensions with virtual EVOQUE valve positioning and longitudinal views of the RH chambers (including RA height and RV depth) are also shown in diastole and systole. Pre-procedural image screening is essential for valve selection. The lower panel illustrates available EVOQUE prosthesis sizes (44–56 mm). CCT is essential for TTVR feasibility, sizing, and procedural planning.

Other orthotopic devices are at earlier stages of development but show promising results. The GATE system, the first dedicated TTVR device, demonstrated feasibility with an 87% procedural success rate in a compassionate-use study, though associated with conduction disturbances and some mortality during follow-up [119]. The LuX-Valve, evaluated in first-in-human and in the TRAVEL trial, achieved high rates of TR reduction to mild-or-less with sustained benefits at 1 year and low complication rates. Its newer iteration, LuX-Valve Plus, allows less invasive jugular access with similar efficacy [120]. Additional systems such as Topaz (TRICURE trial), TriSol, CardioValve (TARGET trial), Intrepid and VDyne are further expanding the therapeutic landscape, although evidence remains limited and largely derived from early feasibility or small cohort studies [121,122,123].

CAVI systems offer a different strategy by placing valves in the superior and/or inferior vena cava to reduce venous backflow and systemic congestion without directly correcting the tricuspid lesion. This approach is usually reserved for patients unsuitable for conventional repair or replacement. Registries have shown improvements in symptoms and QoL despite limited hemodynamic changes, and some evidence suggests potential RV reverse-remodeling [124]. The TricValve system, approved in Europe, achieved a 94% procedural success and significant symptomatic benefit in the TRICUS EURO trial, with sustained results at 1 year in pooled analyses [125]. However, CAVI remains a palliative option, and its long-term prognostic impact remains uncertain. Other heterotopic devices, such as Trillium, are under early clinical investigation.

Overall, TTVR is a rapidly evolving field with expanding device options and encouraging early outcomes. Orthotopic systems represent a more definitive TR treatment, whereas heterotopic approaches provide a simpler alternative for highly selected ineligible patients. Nevertheless, anatomical constraints and limited long-term data continue to mandate careful patient selection.

#### 6.3.4 Management of CIED-Related Tricuspid Regurgitation

In patients with CIED-related TR, the initial step is to determine whether the regurgitation is causally related to the lead (CIED-induced TR) or merely incidental (CIED-associated TR). This requires combined TEE and CT evaluation to assess lead–tricuspid valve interaction. Approximately 35% of patients undergoing TTVR have pre-existing transvalvular CIED leads [115] and CIED implantation is an established risk factor for TR progression [126]. Although echocardiographic guidance prompted lead repositioning in 54% of cases in one study, it is not routinely performed [127].

Mechanisms such as leaflet impingement, subvalvular entanglement, or RV dys-synchrony may be amenable to electrophysiological interventions, including lead repositioning, transvenous lead extraction (TLE), or conversion to leadless or CS pacing systems. However, TLE should be undertaken cautiously, especially when leads are intertwined with the subvalvular apparatus, as this may exacerbate TR. Clinically relevant TR worsening after TLE has been reported in approximately 2.5% of patients, although rates as high as 27% have been described [128,129]. Severe extraction-related TV damage may render subsequent T-TEER or TTVR no longer feasible [63]. Risk factors for worsening TR after TLE include implantation duration >10 years, age >75 years, extraction of ≥2 leads, female sex, sheath-assisted extraction, and operator experience. Conversely, other investigators reported improvement to “normal” TR function after TLE in 9.7% of patients [130]. No prospective data currently support TLE in the absence of lead malfunction or infection.

When electrophysiological strategies are not feasible, transcatheter options such as T-TEER, TTVR, or CAVI can be considered, although repair is often technically challenging and valve replacement may entail risks of lead damage or “jailing”. The main concerns during TTVR are lead infection and dysfunction after lead jailing. Infection risk should be assessed using validated scores such as PADIT [131], which incorporates procedural history, age, renal dysfunction, and immunocompromised status. Lead dysfunction was not reported in the earliest TTVR experience, while the VIVID registry [112] showed that dislodgment or dysfunction of permanent pacemaker occurred in 3 of 28 patients (11%). However “jailing” between a rigid annulus in post-surgical procedures may not be equivalent to caging a lead against the more flexible native annulus. Lead dysfunction is a relevant problem when alternative pacing options are not available, although recent advances in CIED techniques now offer a wide variety of options: His-bundle, epicardial, and CS, leadless pacing, and subcutaneous/extravascular ICD systems [63]. More recently, His-bundle pacing from the RA has been reported as an option [132].

Transvalvular CIED leads may represent a technical challenge for repair techniques, particularly when located near or within the regurgitant jet, causing severe leaflet(s) impingement or fixation. However, a transvalvular lead should not be considered a contraindication to T-TEER. Careful assessment of lead mobility, leaflet interaction, and the spatial relationship with the regurgitant jet is essential to procedural feasibility. When sufficient mobility and redundancy are present, the lead can be displaced away from the regurgitant jet using a steerable catheter; if fixed, clip implantation adjacent to or on either side of the lead may still allow adequate leaflet grasping. T-TEER has been shown to be feasible and safe in this setting. In TRILUMINATE Pivotal, no lead dysfunction occurred among 98 patients with pre-existing transvalvular leads [133]. Although CIED-induced TR required fewer implanted clips and longer procedures, procedural success, TR reduction, functional and symptomatic improvement were comparable to those in patient without leads, with greater symptomatic benefit. By contrast, the PASTE registry identified transvalvular CIED-leads as an independent predictor of residual ≥moderate TR after Pascal implantation [92]. Notably, the TRILUMINATE excluded patients with severe lead impingement or unsuitable anatomy, whereas in the bRIGHT-EU post-approval study, 110 of 511 patients had endocardial leads, 80.9% with CIED-induced TR, and treatment success was achieved in approximately 71% [134]. Management of lead-related TR requires a multidisciplinary Heart Team including experienced electrophysiologists.

#### 6.3.5 Matching Patient and Procedure

Optimal TTVI outcomes rely on careful matching between patient characteristics and device selection. Decision-making should be multidisciplinary, with the Heart Team integrating the underlying etiology and mechanism of regurgitation, detailed valve anatomy, RV performance and technical feasibility, including device deliverability and anatomical eligibility [135].

In PTR, TEE is the first-line imaging modality, allowing comprehensive assessment of regurgitation severity, mechanism, annular dimensions, coaptation gap, and RV function. When focal abnormalities such as leaflet prolapse, flail, or PM rupture are identified, T-TEER is generally the preferred strategy. Conversely, in the presence of leaflet perforation, restriction, calcification, or insufficient tissue, CCT becomes essential to further characterize annular size and the spatial relationships between the valve, RV, coronary arteries, and venae cavae, thereby guiding the choice between TTVR and CAVI.

In STR, distinguishing atrial from ventricular forms is crucial. In patients with A-STR, particularly when associated with recent-onset AF, rhythm-control strategies such as catheter ablation may reduce TR severity. In V-STR, RHC is important to define pulmonary hemodynamics. Fixed precapillary PH generally limits treatment to medical therapy, whereas in its absence, RV function and RV–PA coupling become central in guiding intervention. In all cases, comprehensive imaging with both TEE and CCT is essential to define anatomical suitability.

Based on this integrated evaluation, treatment can be broadly matched to anatomy and physiology. T-TEER is favored in the presence of moderate annular dilation, a coaptation gap <8.5 mm, a coaptation depth <10 mm, and preserved RV function. When annular dilation is the predominant mechanism, with larger coaptation gaps (>8.5 mm) but still adequate RV performance, annuloplasty may be more appropriate. TTVR is generally preferred in patients with large annuli and significant coaptation defects when anatomy is suitable for device implantation, regardless of coaptation depth. By contrast, patients with advanced disease—characterized by severe annular dilation, large coaptation gaps, RV dysfunction, and unfavorable anatomy—are often limited to CAVI or palliative management.

A key objective in this pathway is the identification of patients who are most likely to benefit from intervention, thereby avoiding futility. Ideal candidates should not exhibit advanced frailty, severe RV systolic dysfunction, fixed precapillary PH, and a TAPSE/PASP ratio <0.406. They should have at least moderate-to-severe TR despite optimized medical therapy, including diuretics and SGLT2 inhibitors, together with evidence of worsening HF, such as systemic congestion or reduced cardiac output. Advanced stages of HF with signs of irreversible right-sided dysfunction should generally be excluded from intervention.

Additional studies using invasive measurements of pulmonary pressures suggest that direct hemodynamic assessment may further refine risk stratification and improve outcome prediction. However, robust data correlating RV functional parameters with outcomes after TTVR are still lacking [136].

In summary, T-TEER remains the most frequently adopted approach, particularly in STR, but is limited by large coaptation gaps, suboptimal leaflet anatomy, imaging constraints, and the presence of intracardiac leads. Annuloplasty offers a more physiological correction but is influenced by RV remodeling and anatomical proximity to the RCA. Orthotopic TTVR provides a more definitive solution but is restricted by strict anatomical and technical requirements, including annular size, RV geometry, and access considerations. At the extreme end of the spectrum, CAVI represents a simplified and often the only viable option for patients with advanced disease, although it does not directly address the underlying valvular pathology.

#### 6.3.6 Post-Procedural Antithrombotic Therapy Following T-TEER and TTVR

Peri-procedural and post-procedural antithrombotic therapy is essential to prevent thromboembolic complications related to TTVI. The Tricuspid Valve Academic Research Consortium (T-VARC) consensus document [14] standardizes definitions for venous thromboembolic complications, pulmonary embolism, and device-related thrombosis (DRT). According to T-VARC, thromboembolic and bleeding events are classified as acute (<24 h) or subacute (>24 h to <30 days), early (30 days to one year) and late (>1 year) after the index TTVI procedure.

RH transcatheter interventions carry an increased thrombotic risk because of low-flow venous circulation. Randomized evidence is lacking. Current expert consensus recommends procedure-specific antithrombotic therapy. In patients undergoing T-TEER without another indication for anticoagulation, single antiplatelet therapy (SAPT) is generally considered sufficient, whereas TTVR generally requires anticoagulation for at least 3 months because of the higher risk of prosthetic valve thrombosis during the endothelialization phase [137]. AF is highly prevalent among TTVI candidates (90% in TRILUMINATE and 97% in TRISCEND II) [36,118]. Patients with AF already receiving direct oral anticoagulants (DOACs) or vitamin K antagonists (VKAs) should continue lifelong oral anticoagulation (OAC). Only two DRT cases (0.7%) were reported in TRILUMINATE and none among the 511 patients in the bRIGHT registry [84], supporting SAPT after T-TEER in patients without another indication for OAC. Hoebrand et al. [138] demonstrated a favorable risk-benefit profile for patients with a concomitant indication for OAC. Despite elevated thromboembolic and bleeding risk scores, only one case of major bleeding was reported in 78 patients undergoing T-TEER while on DOACs. Patients treated with DOACs showed a significant reduction of major bleeding events over VKAs (*p* = 0.044) but no difference in mortality. These findings should be interpreted in light of the low proportion of patients receiving DOACs in the TRILUMINATE trial (2%). Because annuloplasty is also a repair technique, the same antithrombotic strategy appears appropriate [101,102,104,105,106,107,108,109]. Compared with T-TEER, DRT is substantially more frequent after TTVR (27%) [139]. The VIVID registry [112] showed 26.1% of DRT after TTVR (valve-in-valve or valve-in-ring) with Melody (Medtronic) or Sapien (Edwards Lifesciences) prostheses after a median of 15.9 months. The majority of DRT were subacute and about one-third were peri-procedural, and most patients were not on OAC. These findings are consistent with long-term surgical data, in which prosthetic valve thrombosis is considerably more common in right-sided bioprosthetic valves (up to 30%) and mechanical valves (4–20%) [140,141]. Thrombosis is related not only to RH rheology but also to the large prosthetic valve surface area. For surgical bioprosthetic valves, therapy with either DOACs or VKAs is suggested for 3–6 months after TVR [12]. In a multicenter study of the LuX-Valve system, in which all patients received OAC for at least six months, only one case of DRT (0.8%) was reported at one-year follow-up [120]. Nevertheless, the high hemorrhagic risk of patients treated with TTVR deserves careful consideration because 10.4% of patients experienced major bleeding in the TRISCEND II trial and similar rates have been reported for the LuX-Valve [118,120]. Based on current expert consensus, OAC for at least 3 months after TTVR is recommended together with close multimodality imaging follow-up in order to detect subclinical DRT, although dedicated clinical trials are still needed [142]. Antithrombotic management after CAVI is based on limited available evidence. All patients were treated with OAC in the TRICUS EURO trial (94% were in AF) [125], whereas observational data support either 3 months OAC followed by dual antiplatelet therapy (DAPT) in patients without AF (<22%) or DAPT for 4 weeks after discharge [143]. Intravenous unfractionated heparin to maintain an activated clotting time >250–300 s is recommended to prevent intraprocedural thromboembolic events [137]. Pending RCTs, antithrombotic therapy after TTVR in patients without a pre-existing indication for OAC should be individualized according to the device type, bleeding risk, and multimodality imaging findings suggestive of DRT.

## 7. Limitations and Evidence Gaps

Despite consistent improvements in TR severity and QoL, RCTs of T-TEER and TTVR have not demonstrated a significant survival benefit. This is confirmed by recent meta-analyses comparing TTVI with optimal medical therapy [144,145]. Even though pooled analyses including observational studies (13 trials including 6732 patients) showed lower mortality and HFH after TTVI, these benefits were not confirmed when only RCTs were analyzed, suggesting that residual confounding and selection bias substantially influence observational evidence [146]. Moreover, currently available RCTs remain underpowered for hard clinical endpoints because of limited sample size, lower-than-expected event rates, and relatively short follow-up (3–30 months; Table 1 (Ref. [36,85,86,88,91,92,115,117,118,120,147])). Clinical benefit after TTVI appears early after the procedure, whereas prognostic differences emerge during longer follow-up. In TRILUMINATE, HFH became significantly reduced after 2 years and decreased by 75% after 3 years, while a similar reduction was observed at 1 year in the TriCLASP study [85,86,148,149]. Whether this will translate into long-term survival benefit remains uncertain. In addition, the long-term durability of TTVR remains largely unknown, particularly considering the structural degeneration observed in surgical bioprosthetic valves [140,141].

**Table 1.** **Main T-TEER and TTVR trials**.

Trial name	Device	Design	Sample size (n)	TR severity	Primary endpoints	Outcomes	Follow-up (months)
TRILUMINATE Pivotal [36]	TriClip	RCT: T-TEER vs medical therapy	350 (172 T-TEER)	Severe symptomatic TR	Hierarchical composite endpoint (ACM or TV surgery, HFH, ↑KCCQ by at least 15 points)	-No significant mortality and HFH benefit at 1 year -↑KCCQ -TR ≤ moderate: 87% -↓NYHA class, 6MWD	1 and 12
TRILUMINATE Single-Arm [85]	TriClip	Prospective, multicentre, single-arm study	85	Moderate or higher TR	• Primary efficacy endpoint: ↓TR by at least 1 grade at 30 days • Primary safety endpoint: composite of adjudicated MAEs at 6 months	• 30-day outcomes: -Procedural success: 100% -TR ≤ moderate: 86% • 6-month outcomes: -NYHA I or II: 86% -↑KCCQ by 18.4 points -↑6MWD: 61.9 m -ACM: 5% -Major bleeding: 11%	1 and 6
TRILUMINATE Extension [86]	TriClip	Long-term follow-up of the treated cohort	98	Severe symptomatic TR	Same as TRILUMINATE Single-Arm	- HFH reduction at 2- and 3-year follow-up	36
bRIGHT [88]	TriClip	Prospective real-world post-approval study	511	Massive or torrential TR	• ≥1-grade TR reduction at discharge • Composite of adjudicated MAEs • Clinical status (NYHA, KCCQ-OS)	-30-day outcomes: -≥1-grade TR reduction: 77% -in-hospital mortality: 0.8% -MAEs: 2.7% -1-year outcomes: TR ≤ moderate: 81% -NYHA I or II: 75% -ACM: 15.1%	1 and 12
CLASP TR Early Feasibility Study [91]	PASCAL	Single-arm, multicenter, prospective study	65	Severe to torrential TR	• Composite of MAEs at 30 days and 1 year • Performance endpoints: -Implant success -Procedural success (≥1-grade TR reduction) • Clinical endpoints: -NYHA, KCCQ, 6MWD, ACM, HFH	• 30-day outcomes: -Implant success: 90.8%; MAEs: 9.2%; Major bleeding: 7.7%; ACM: 3.1%; TR ≤ moderate:70% • 1-year outcomes: -MAEs: 16.9% -Major bleeding: 9.2% -Major access site and vascular complications: 3.1% -ACM: 10.8% -HFH: 18.5% -TR ≤ moderate: 86% -NYHA I or II: 92% -↑Mean 6MWD: 94 m -↑Mean KCCQ score: 18 points	1 and 12
TriCLASP Study [147]	PASCAL	Single-arm, multicenter, prospective study	74	Severe symptomatic TR	• Composite of MAEs at 30 days and 1 year • Performance endpoints -Implant success -Procedural success (≥1-grade TR reduction) -Clinical and echocardiographic endpoints	-30-day outcomes: -TR ≤ moderate: 90%; MAEs: 3%; CVM: 1.5%; Major bleeding: 1.5%; ↓NYHA class: 54%; ↑6MWD: 38.2 m; ↑Mean KCCQ: 13.4 -1-year outcomes: -TR ≤ moderate: 87.7%; MAEs: 12.7%; -yearly HFH reduced by 75%; ↓NYHA class: 74.5%; ↑mean 6MWD: 29.4 m; ↑mean KCCQ: 8.3	1 and 12
PASTE [92]	PASCAL	Multicenter, prospective observational study	1059 (First-generation PASCAL: 570; PASCAL Precision: 489)	Severe or higher grade TR	-T-VARC procedural success and clinical efficacy. -ACM and HFH -Comparison between PASCAL and PASCAL Precision	-Procedural success: 85% -TR ≤ moderate: 87%; ACM: 14%; HFH: 16% -NYHA class, 6MWD and KCCQ improvement -PASCAL Precision: higher procedural efficacy than first-generation PASCAL	12
TRISCEND I [115,117]	EVOQUE	Prospective, observational single-arm, multicenter study	176	Severe or higher grade TR	Composite of MAEs at 30 days and 1 year • Performance endpoints: -Implant success -Procedural success (≥1-grade TR reduction) -Clinical and echocardiographic endpoints	-Device success: 94.4% -Procedural success: 93%; clinical success: 77.1% 1-year outcomes: -MAEs: 30.2%; CVM: 9.4%; Major bleeding: 25.5%; TR ≤ mild: 97.6% -NYHA I or II: 93.5% -ACM: 9.1% -HFH: 10.2% -↑KCCQ 25.7 points -↑mean 6MWD 56.2 m	1 and 12
TRISCEND II [118]	EVOQUE	Multicenter RCT: EVOQUE vs medical therapy	400 (TTVI: 267; Control: 133)	Severe or higher grade TR	Primary safety endpoint; hierarchical composite efficacy endpoint: ACM or TV Surgery, VAD or transplant, HFH, health status improvement	-Primary safety endpoint met -TR ≤ mild: 95% -Health status improvement (NYHA class, 6MWD) -No effect on HFH -No significant difference for ACM	18
TRAVEL [120]	LuX-Valve	Single-arm, multicenter, prospective observational study	126	Severe symptomatic TR	-Primary endpoint: ACM and HFH at 1 year -Safety and efficacy	-ACM: 10.3% -HFH: 4.8% -Procedural success: 95.2% -Major bleeding: 14.3% -Health status improvement (NYHA class, 6MWD)	12

TR, tricuspid regurgitation; RCT, randomized clinical trial; MAE(s), major adverse event(s); ACM, all-cause mortality; HFH, heart failure hospitalization; CVM, cardiovascular mortality; NYHA, New York Heart Association; 6MWD, 6-minute walk distance; KCCQ-OS, Kansas City Cardiomyopathy Questionnaire Overall Summary Score; T-TEER, transcatheter tricuspid edge-to-edge repair; TTVR, transcatheter tricuspid valve replacement; T-VARC, Tricuspid Valve Academic Research Consortium; VAD, ventricular assist device. ↑ indicates increase; ↓ indicates decrease.

Head-to-head comparisons between T-TEER and TTVR are lacking, and no RCT has directly evaluated annuloplasty against T-TEER in A-STR patients. Meta-analyses suggest that T-TEER has a more favorable safety profile than TTVR, with lower rates of major bleeding and permanent pacemaker implantation [144,145]. However, comparisons between repair and replacement strategies are complicated by major methodological differences. T-TEER trials enrolled larger populations (1966 vs 569 patient undergoing TTVR, Table 1) with longer follow-up and consistently demonstrated improvements in symptoms, QoL, NYHA class, KCCQ and exercise capacity, whereas TTVR studies mainly provide evidence of procedural efficacy and TR elimination in smaller and more advanced populations. Patients undergoing TTVR are generally older, more frequently women, and more likely to present with chronic AF, transvalvular CIED leads (36%) and advanced stages of disease characterized by cardio-hepatic and cardio-renal syndromes [146]. Consequently indirect comparisons between repair and replacement strategies should be interpreted cautiously, highlighting important evidence gaps.

Several factors may explain discrepancies in TTVI trials results. First, TR is a highly heterogeneous disease with different etiologies, phenotypes, and responses to treatment. Real-world studies suggest better outcomes in A-STR than in V-STR, probably reflecting earlier disease stage with fewer comorbidities, preserved RV function, and more favorable anatomy rather than treatment effect alone. However, important gaps remain regarding A-STR subtypes (paroxysmal, persistent or chronic AF) and standardized phenotypic classification (e.g., EuroTR registry did not include RV contractility and diameter data for all patients).

Selection bias across trials remains substantial. Conventional surgical risk models (STS and TRI-SCORE) are commonly used despite their limited calibration in TTVI populations. Dedicated TTVI risk models, including TRIVALVE and EuroTR, provide better prognostic discrimination, but still require prospective external validation before routine incorporation into guideline-directed patient selection or trial eligibility criteria [52,53].

Assessment of RV function also remains inconsistent. TRILUMINATE did not include TAPSE or RV dilatation among inclusion criteria, whereas TRISCEND II excluded patients with TAPSE <10 mm, and TRICUS-EURO adopted a cut-off of <13 mm, resulting in markedly different baseline RV contractility across studies. Mean baseline TAPSE in TRILUMINATE, TRISCEND II and TRICUS-EURO were 14.8 mm, 16 mm, and 18 mm, respectively [85,118,125]. Furthermore, RV-PA coupling parameters such as the TAPSE/PASP ratio have not been incorporated into any RCT despite their prognostic value.

TR proportionality has not yet been prospectively evaluated in TTVI trials. The EROA/RVEDD ratio has recently been proposed as a prognostic marker [60]. Patients with RV-dominant disproportionate TR (EROA/RVEDD <0.110) appear to have advanced RV remodeling and worse prognosis despite effective TR reduction, probably identifying a patient subset with late disease stage, who may experience symptom improvement without clear survival impact. The mean baseline EROA/RVEDD in the TRILUMINATE Pivotal (0.12) was close to this threshold, suggesting that a substantial proportion of the study population may already have had advanced RV remodeling relative to TR severity. Together with selection bias, fewer-than-expected events, and inadequate statistical power for hard endpoints due to sample size, this may partly explain the absence of a demonstrated mortality benefit.

These findings raise the question of whether symptom relief, QoL and functional recovery should themselves justify intervention in elderly frail patients with advanced TR. Although these patient-centered outcomes are clinically meaningful, they should be balanced against procedural risk, treatment durability, and the likelihood of futility, and complemented by formal cost-effectiveness analyses using quality-adjusted life years (QALYs). Dedicated cost-effectiveness analyses remain limited [150]. Thus, QoL improvements should not replace assessment of mortality and HFH.

The prognostic impact of PH has not been fully addressed because patients with severe PH were excluded from RCTs. In the large EuroTR registry (N = 1230 patients), invasive mPAP >46 mmHg independently predicted mortality after T-TEER, whereas TR reduction and symptomatic improvement were not influenced by PH severity [151]. Pre-capillary and post-capillary PH showed similar results. In contrast with RHC assessment, the previously described echocardiographic cut-off of 30 mmHg was not associated with HF-free survival in this population [46].

Future RCTs should therefore incorporate proportionality indices, RV reverse-remodeling, invasive hemodynamic assessment, RV-PA coupling, and validated TTVI-specific risk scores to improve patient selection.

Another unresolved issue concerns the optimal timing of intervention. Available clinical studies suggest that most deaths were not device- or cardiac-related in both observational and RCTs, occurring predominantly in older and frailer patients, with ascites, severe CKD and a very high EuroSCORE II and TRI-SCORE before TTVI [125,143]. This underscores the importance that end-stage patients may not benefit from interventional procedures. A precise definition of end-stage disease and patient frailty is lacking. Current evidence suggests that intervention before irreversible RV remodeling and end-stage right HF may improve long-term outcomes, whereas advanced frailty and multi-organ dysfunction are associated with limited benefit despite successful TR reduction.

Finally, important evidence gaps remain in patients with CIED-related TR. No prospective study has directly compared T-TEER with TTVR in this setting. Valve-sparing pacing strategies, including RA His-bundle pacing, and the long-term impact of lead jailing remain poorly investigated, particularly because patients with severe lead impingement were excluded from major T-TEER trials. Prospective data regarding the long-term impact of clip implantation adjacent to transvalvular leads on lead function and the feasibility of future lead extraction are also lacking.

Temporary superior vena cava balloon occlusion [152] and adjunctive RV unloading strategies (temporary RV mechanical support using Impella RP, aggressive pre-procedural diuresis or ultrafiltration, ventricular-assist maneuvers, and patient positioning) warrant further investigation in patients with large coaptation gaps.

Emergency T-TEER has been described in isolated cases of acute TR with RV shock. Valle et al. [153] described a successful treatment in a patient with hypertrophic cardiomyopathy. Evidence remains limited to isolated case reports, and no guideline recommendations currently address this scenario.

## 8. Conclusions

During the last few years, significant advances have been made in the scientific evidence regarding the diagnostic evaluation, clinical classification, and prognosis of significant TR. The paradigm of TR is shifting from late palliative management to earlier, phenotype-driven intervention. However, gaps remain concerning the optimal timing of surgical or percutaneous treatment. Pending adequately powered prospective studies, to determine whether a true “point of no return” exists, beyond which treatment may no longer provides prognostic advantage, the currently available evidence allows the construction of a proposed late-stage TR phenotype. Fig. 5 integrates these findings into a conceptual framework illustrating disease progression, the potential therapeutic window, and the clinical profile of patients in whom intervention may become predominantly palliative rather than prognostic.

**Fig. 5.**
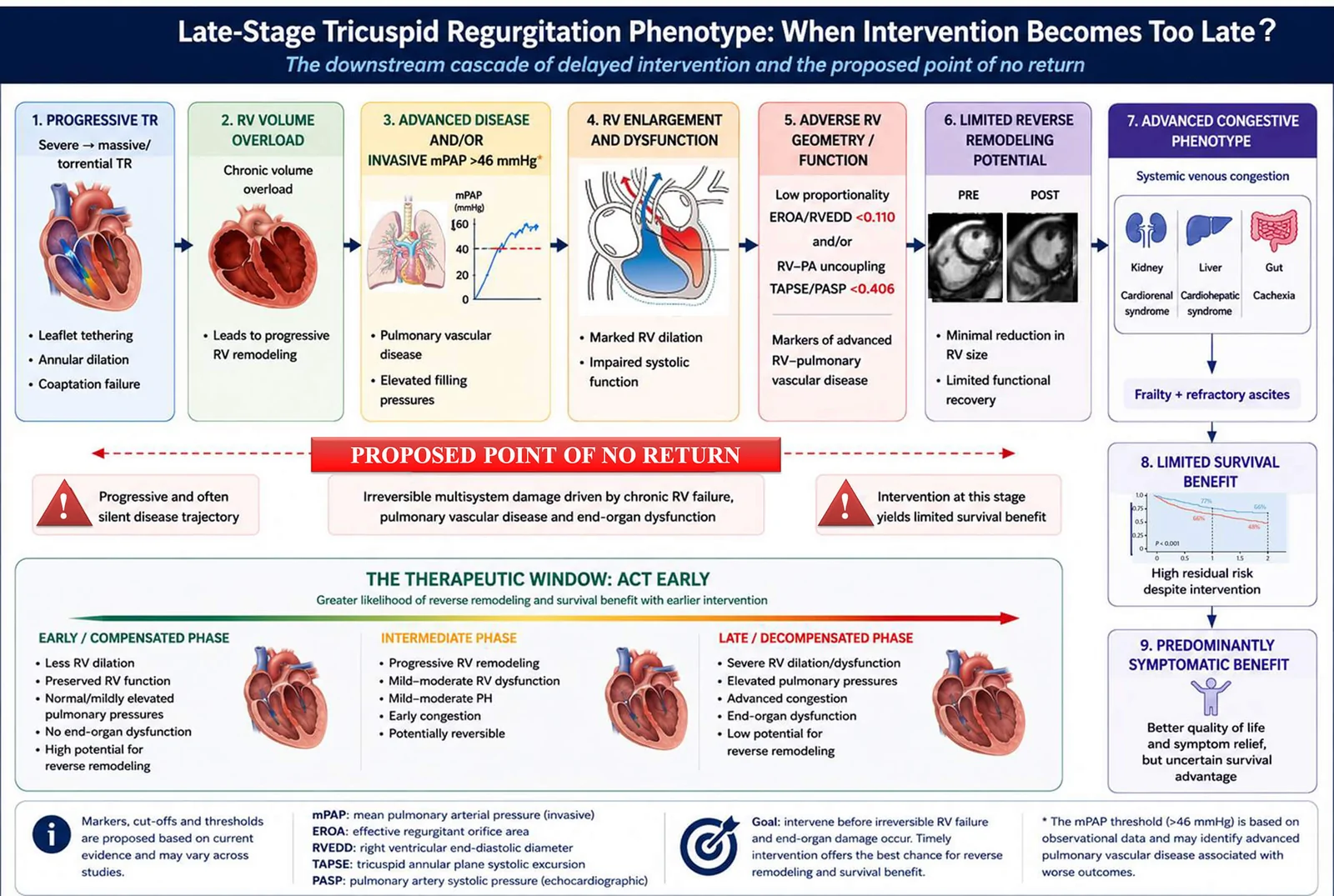
**Proposed late-stage tricuspid regurgitation phenotype and therapeutic window for intervention**. Progressive severe TR leads to RV volume overload, adverse remodeling, pulmonary vascular disease, and systemic venous congestion. As disease advances, unfavorable hemodynamic markers—including low TR proportionality (EROA/RVEDD <0.110), impaired RV-PA coupling (TAPSE/PASP <0.406), and elevated invasive mPAP (>46 mmHg)—may identify patients with limited potential for RV reverse-remodeling after intervention. Beyond the proposed “point of no return”, multiorgan involvement (cardiorenal/cardiohepatic syndrome, cachexia, refractory ascites, and frailty) may limit the likelihood of survival benefit, although symptomatic improvement may still occur. The lower panel illustrates the proposed therapeutic window, where earlier intervention may maximize reverse remodeling and long-term outcomes. Thresholds are derived from current observational evidence and should be considered hypothesis-generating.

Future adequately powered RCTs with longer follow-up are required to identify the optimal timing of intervention, define thresholds for RV dysfunction and PH, compare repair and replacement strategies, and determine the long-term durability of transcatheter devices.
